# RAGE signaling during tobacco smoke-induced lung inflammation and potential therapeutic utility of SAGEs

**DOI:** 10.1186/s12890-022-01935-x

**Published:** 2022-04-26

**Authors:** Kelsey M. Hirschi-Budge, Kary Y. F. Tsai, Katrina L. Curtis, Gregg S. Davis, Benjamin K. Theurer, Anica M. M. Kruyer, Kyle W. Homer, Ashley Chang, Pam M. Van Ry, Juan A. Arroyo, Paul R. Reynolds

**Affiliations:** 1 Lung and Placenta Laboratory, Department of Cell Biology and Physiology, Brigham Young University, Provo, UT USA; 2 Department of Chemistry and Biochemistry, Brigham Young University, Provo, UT USA

**Keywords:** RAGE, Tobacco, Lung, Inflammation

## Abstract

**Background:**

Smoke exposure culminates as a progressive lung complication involving airway inflammation and remodeling. While primary smoke poses the greatest risk, nearly half of the US population is also at risk due to exposure to secondhand smoke (SHS).

**Methods:**

We used WT, RAGE−/− (KO), and Tet-inducible lung-specific RAGE overexpressing transgenic (TG) mice to study the role of RAGE during short-term responses to SHS. We evaluated SHS effects in mice with and without semi-synthetic glycosaminoglycan ethers (SAGEs), which are anionic, partially lipophilic sulfated polysaccharide derivatives known to inhibit RAGE signaling. TG Mice were weaned and fed doxycycline to induce RAGE at postnatal day (PN) 30. At PN40, mice from each line were exposed to room air (RA) or SHS from three Kentucky 3R4F research cigarettes via a nose-only delivery system (Scireq Scientific, Montreal, Canada) five days a week and i.p. injections of PBS or SAGE (30 mg/kg body weight) occurred three times per week from PN40-70 before mice were sacrificed on PN70.

**Results:**

RAGE mRNA and protein expression was elevated following SHS exposure of control and TG mice and not detected in RAGE KO mice. Bronchoalveolar lavage fluid (BALF) analysis revealed RAGE-mediated influence on inflammatory cell diapedesis, total protein, and pro-inflammatory mediators following exposure. Lung histological assessment revealed indistinguishable morphology following exposure, yet parenchymal apoptosis was increased. Inflammatory signaling intermediates such as Ras and NF-κB, as well as downstream responses were influenced by the availability of RAGE, as evidenced by RAGE KO and SAGE treatment.

**Conclusions:**

These data provide fascinating insight suggesting therapeutic potential for the use of RAGE inhibitors in lungs exposed to SHS smoke.

## Background

Exposure to cigarette smoke is among the top ten contributors to the worldwide health burden and a key cause of preventable deaths [1]. There have been various societal efforts aimed at curbing smoking popularity; however, indicators suggest ongoing increase [2, 3]. Cigarette smoke is prevalent—nearly 50% of the American population is regularly exposed and nearly a fifth of America’s youth live with a smoker in the home [4]. Risk of chronic diseases such as chronic obstructive pulmonary disease (COPD), a disease characterized by inflammation and irreversible airflow obstruction, comprises a notable aspect of overall health burden [5]. COPD is the third leading cause of death in the United States [6] and its severity and incidence continue to increase. COPD diagnoses doubled in the past decade and a half, and in 2016, over 150,000 persons died in the U.S. alone [7, 8]. American COPD statistics are staggering: 8 million outpatient visits, 1.5 million emergency room visits, and 672,000 hospitalizations 2008 [7]. In 2010, the economic burden associated with COPD was approximately $50 billion, of which $30 billion was in direct costs. Exacerbations of COPD triggered by primary and secondhand exposure annually accounts for notable mortality and worsened quality of life. The current research endeavor was not a chronic study of smoke exposure that culminates in COPD pathogenesis; rather, it’s acute nature was sought to identify short term inflammatory mechanisms stemming from exposure. This research, and similar research of others, may be helpful by providing insight with potential relevance to long term exposure.

Smoking causes acute inflammation and injury; however, long-term exposure is the leading risk factor for the development of COPD [9, 10]. Despite abundant COPD diagnoses in current and/or former smokers, the disease is also detected among populations of never smokers [11]. In fact, more recent attention has been appropriately applied to patients residing in areas where exposure to secondhand smoke (SHS) prevails [12, 13]. Former smokers often maintain their residence with active smokers and therefore may experience COPD later in life, likely due to persistent exposure to passive smoke. As a general theme, smokers diagnosed with moderate COPD stemming from first or secondhand exposure experience altered gene expression including genes implicated in regulating transcription, cellular growth, and the remodeling of extracellular matrix [14]. Gene products differentially altered by prolonged cigarette smoke exposure maintain general functions including inflammatory leukocyte recruitment, cytokine elaboration, cell turnover, and matrix degradation. As such, it is critical to examine how genes influence the disease so that precise mechanisms by which passive and active cigarette smoke contributes to acute lung inflammation can be identified. Our present focus was on acute exposure because the acute activation of signaling pathways may be a determinant that becomes prolonged in long-term disease progression. Complimentary chronic studies remain essential.

Diverse cell types have been identified as cells that express receptors for advanced glycation end-products (RAGE). RAGE is a cell-surface receptor of the immunoglobulin superfamily detectible in cells such as endothelial and vascular smooth muscle cells, fibroblasts, macrophages, and epithelium [15]. RAGE was first described as a pulmonary-specific progression factor involved in responses coordinated by irreversibly glycated proteins called advanced glycation end-products (AGEs) [16]. Studies have previously demonstrated that highly reactive glycation products are contained in cigarette smoke and they function to rapidly cause AGE formation both in vitro and in vivo [17, 18]*.* Cigarette smokers and nonsmokers express altered levels of serum AGEs as well as apolipoprotein B-linked AGEs [17], and AGEs are present at higher levels in various body tissues of smokers compared to nonsmokers [18]. Interestingly, a role for AGEs is further reinforced in experiments involving the elimination of smoke-induced AGE formation when samples are exposed potent inhibitors of AGE formation such as aminoguanidine.

RAGE has further been established as a pattern recognition receptor that perpetuates signal transduction pathways following ligation with calcium-binding S100/calgranulins, amyloid-β-peptide, and HMGB-1. These differentially impacted signaling cascades involve MAP kinases (ERK, JNK, and p38), NF-κB, reactive oxygen species (ROS), and TNF/IL-1 [19, 20] and their culminating influence on inflammation [21]. Unmitigated engagement of RAGE by its ligands does not burn out; rather, it stems into prolonged inflammatory effects coincident with severe tissue injury [22].

RAGE expression increases as a direct result of ligand accumulation [16] and RAGE-ligand interaction contributes to diverse pathological processes [19, 20]. Because RAGE ligation up-regulates RAGE and its ligands with “feed-forward” magnification [22, 23], smoke-mediated AGE generation most likely initiates a cascade resulting in enhanced local RAGE signaling. Our previous work demonstrated that SHS induces the expression of RAGE and its ligands [24–26]. One such prior publication highlighted how acute SHS exposure of WT mice enhanced RAGE expression, RAGE signaling molecules, and inflammatory cytokines in WT mice and how loss of RAGE impacted inflammation [25]. Others have also demonstrated a role for RAGE as a key regulator of inflammation in lung disease. For example, abrogation of RAGE signaling using *RAGE* knockout mice attenuated pulmonary ischemia and reperfusion injury [27], elastase-induced emphysema [28], and significantly protected against smoke-induced airway inflammation [29, 30]. A link between RAGE and lung inflammation was further supported by data that implicated RAGE polymorphisms in lung disease progression [31]. Together, these studies demonstrate that RAGE is a key modulator of inflammation, particularly in the context of smoke exposure and lung injury.

The current research endeavor compared short-term SHS exposure in three mouse models: RAGE knock out (KO), conditional lung-specific RAGE overexpressing transgenic (TG), and controls. Acute exposure was selected in order to characterize short-term effects in exposed animals. We demonstrate a role for RAGE in orchestrating inflammatory responses and physiological compromise following exposure. As a means of abrogating RAGE, we provide proof of principle that SAGEs ameliorate SHS-induced phenotypes modulated by inflammatory responses.

## Methods

### Mice and secondhand smoke exposure

Female WT mice (obtained from Jackson Laboratories; Bar Harbor, ME), RAGE KO mice, and conditional RAGE transgenic mice (TG) that were previously confirmed to have increased RAGE expression in alveolar type II epithelial cells [32, 33], were all generated on a C57BL/6 background. Rodents were supplied with food and water ad libitum in a specific pathogen free facility and maintained on a 12-h light–dark cycle. TG Mice were weaned and fed doxycycline ad libitum to induce RAGE at post-natal day (PN) 30 as described in detail [34]. Commencing on PN40, RAGE KO (lacks RAGE), WT (basal RAGE expression) and RAGE TG (elevated RAGE expression) mice (n = 8 per group) were exposed to SHS generated from 3R4F research cigarettes from Kentucky Tobacco Research and Development Center, University of Kentucky using a nose-only exposure system (InExpose System, Scireq, Montreal, Canada). The system generates a 10 s computer-controlled puff every minute; this mainstream (primary) smoke is cleared from the apparatus and expelled via a dedicated pump so that mice are not exposed to primary smoke. A separate pump procures the side stream smoke and delivers it continually until the next puff without any mixing with mainstream smoke. This cyclic approach ensures time to vacate the primary smoke and steady SHS exposure until the next brief interruption associated with subsequent puffs. Treated mice were exposed to SHS from two cigarettes over 10 min, allowed to equilibrate in room air for 10 min, then exposed to smoke from one cigarette for an additional 10 min so that total SHS exposure totals 20 min per day. This procedure was repeated five days a week from PN40-PN70 and compared to groups of mice (n = 8 per group) that were similarly restrained and exposed to room air. The SHS challenge was tolerated well in terms of toxicity and was delivered at an acceptable level of particulate density concentration according to previously published reports [35]. Pilot projects utilizing these exposure conditions revealed that weekly total particulate density concentration varied between 128 and 147 mg total particulate matter per m^3^ with an average of 132.6 mg/m^3^ over the 30 days of exposure. Where indicated, mice were also administered an i.p. injection of PBS vehicle or 30 mg/kg of SAGEs in PBS (three days a week for four weeks). At the conclusion of the exposure, and in line with previously published protocols, mice were sacrificed and lungs were inflation fixed with 4% paraformaldehyde [34], lavaged to characterize bronchoalveolar lavage fluid (BALF), or resected for isolation of total protein or RNA [33, 34]. All experimental animal studies were approved by the Institutional Animal Care and Use Committee (IACUC) at Brigham Young University and all methods were carried out in accordance with relevant animal guidelines and regulations. The reporting of animal methods was in accordance with ARRIVE guidelines for the reporting of animal experiments.

### Semi-synthetic glycosaminoglycan ethers (SAGEs)

As a means of abrogating RAGE, select experiments incorporate an inflammation modulating sulfated polysaccharide derived from hyaluronic acid (HA) called semi-synthetic glycosaminoglycan ethers (SAGEs). SAGEs used in the current research (GM-0111) were a gift from Dr. Glenn D. Prestwich from the University of Utah and they potently prevent RAGE-ligand binding at nanomolar concentrations [36].

### Protein and RNA characterization

Quantitative real time RT-PCR (qPCR) and immunoblotting were performed for RAGE using conditions already described in detail [37]. RNA was isolated using Trizol reagent (Invitrogen, Grand Island, NY) and RNA concentration was confirmed via optical density. Protein from whole lung was isolated by homogenization with RIPA buffer containing protease inhibitors (Fisher Scientific, Waltham, MA). Total protein was quantified using a BCA Protein Assay Kit (Fisher Scientific) and 20 ug of lung protein was used. Immunoblotting was conducted for RAGE (RnD Systems, Pittsburg, PA, #AF1179) and cleaved caspase-3 (Cell Signaling, #9661L). To determine loading consistencies, membranes were also stripped from antibodies and re-probed utilizing an antibody against actin (Cell Signaling; #4967L). All membranes were then incubated with fluorescent secondary antibodies for 1 h and washed 3X with TBST the next day prior to imaging. Membranes were developed with a Li-COR Odyssey CLx (Li-COR Biosciences, Lincoln, NE) wherein fluorescent densities were determined and comparisons were made between the groups.

### Histology

Lungs from at least four animals per group were fixed in 4% paraformaldehyde, processed, embedded and sectioned at 5 µm thickness [34]. Hematoxylin and eosin (H&E) staining was performed to observe general lung morphology. Alveolar size was estimated via mean linear intercept measurement according to guidelines [38]. At least 10 images were evaluated from each mouse using the public domain NIH Image J program (Version 1.x) [39].

### Ras and NF-κB characterization

A Ras Activation ELISA Kit was used to assess the specific expression of active and inactive Ras (Millipore, Temecula, CA). Samples of total lung lysates were first quantified by BCA assay prior to screening Ras expression in 20 μg aliquots. Ras measurements were conducted in triplicate and compared to EGF-treated HeLa cell lysates as a positive control and in replicates performed without lysates as a negative control. A colorimetric high-throughput FACE assays available from Active Motif was used to screen total and active p65 NF-κB as outlined in the manufacturer’s instructions (Carlsbad, CA). Experiments involved a total of six animals per group.

### Bronchoalveolar lavage fluid (BALF) analysis

On the date of sacrifice, BALF was procured and evaluated as outlined previously [37]. Briefly, the trachea was cannulated with a 20-gauge catheter and PBS was lavaged in accordance with the weight of the mouse prior to surgery and removed. Lavage fluid was centrifuged at 4°C, total cells were counted using a hematocytometer, and cell differential counts were performed. Counting was conducted in triplicate and averaged. Abundance of TNF-α, MIP-2, and IL-1β were obtained using molecule-specific ELISA kits used as directed in the manufacturer’s instructions (Ray Biotech, Inc., Norcross, GA).

### Lung mechanics assessment

Lung mechanics measurements were performed as described in Gilhodes et al. and Devos et al. [40, 41]. These lung mechanics measurements were conducted using the flexiVent FX system (SCIREQ Inc., Montreal Qx, Canada). The instrument was equipped with a FX1 module and a Negative Pressure-Driven Forced Expiration (NPFE) extension for mice run by flexiWare 8.0 software. Mice were anesthetized using an intraperitoneal injection of ketamine-xylazine (100 and 10 mg/kg body weight) in 0.9% sterile saline. Once mice were observed to be in a surgical plane of anesthesia, the trachea was exposed to insert a 22-gauge metal cannula. The mice were then attached to the flexiVent and received an intraperitoneal injection of 0.8 mg/kg body weight pancuronium bromide to prevent spontaneous breathing. The plethysmograph chamber was secured over the mice. Mice were ventilated with a tidal volume of 10 mL/kg with a frequency of 150 breaths/min and an end-expiratory pressure of 3 cmH_2_O. The baseline was recorded, and the following scripts were run three times: Deep Inflation, Snapshot-150, Quick Prime, Negative Pressure-Driven Forced Expiration (NPFE) as detailed by the manufacturer. Mice were euthanized after all the scripts were completed.

### Statistics

Mean values ± S.D. from at least six animals per group were assessed by one and two-way analysis of variance (ANOVA). When ANOVA indicated significant differences, student t tests were used with Bonferroni correction for multiple comparisons. Results are representative and those with p values < 0.05 were considered significant.

## Results

### RAGE expression and signaling during SHS exposure

Mice from each of the three described lines (WT, RAGE KO, and RAGE TG) were exposed to SHS for four weeks (from PN40-PN70). Importantly, RAGE was concomitantly induced in RAGE TG mice from PN30-70 in their diet ad libitum*.* There were no observed effects in non-transgenic mice also fed Dox (not shown) or in RAGE TG mice not treated with doxycycline (not shown).

**Fig. 1:**
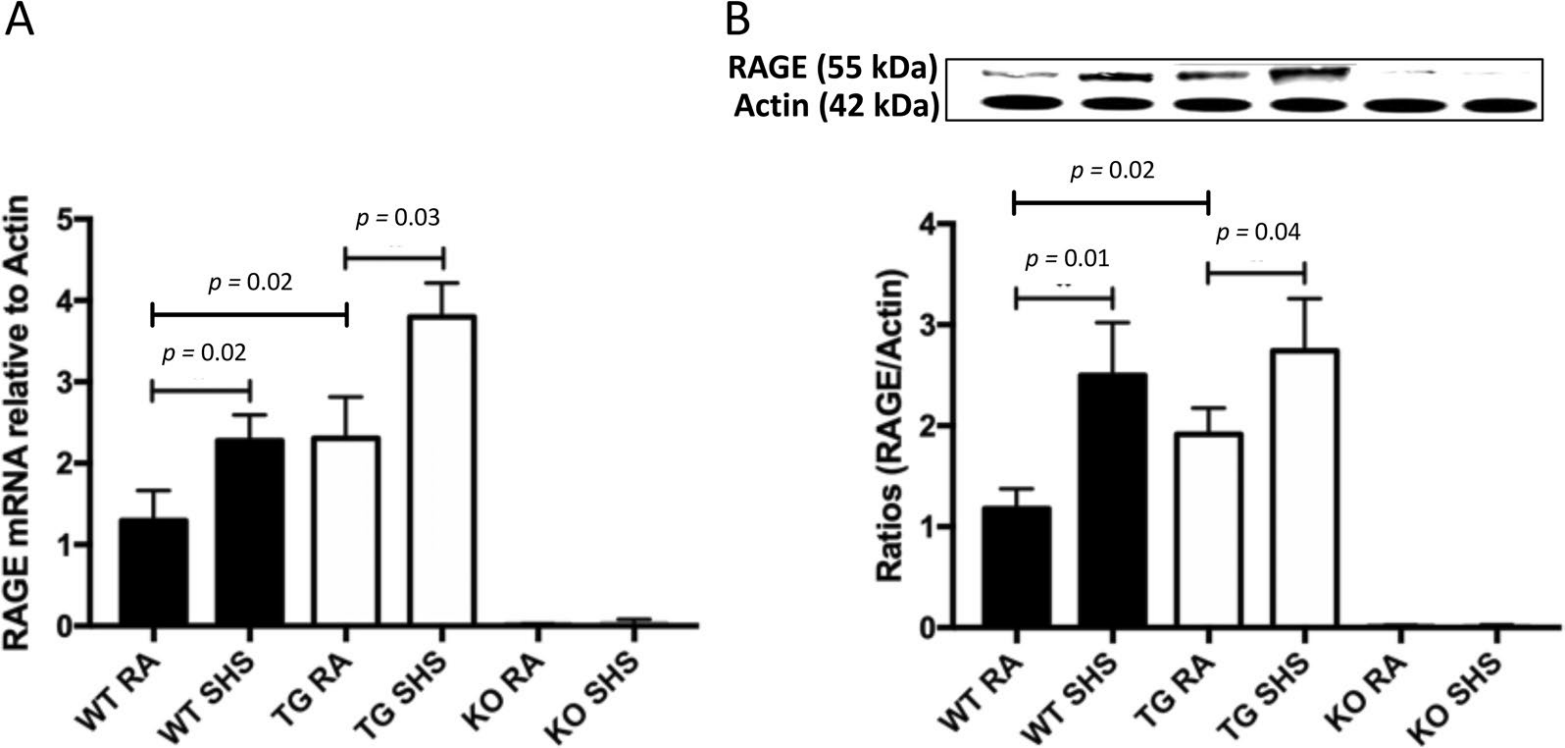
**A** RAGE mRNA expression was elevated in lungs from RAGE TG mice compared to WT controls in the absence of SHS exposure (*p* = 0.02). RAGE mRNA was significantly elevated in WT (*p* = 0.02) and RAGE TG mice (*p* = 0.03) following exposure to SHS compared to room air (RA) controls. Expression of RAGE transcripts were not detected in RAGE KO mice regardless of exposure. The mRNA was normalized to β-actin (n = 6 mice per group) and representative data are shown. **B** Analysis of RAGE protein demonstrated that RAGE TG animals expressed significantly more RAGE protein compared to controls (*p* = 0.02). SHS exposure significantly increased RAGE protein expression in WT (*p* = 0.01) and RAGE TG mice (*p* = 0.04) compared to RA controls while RAGE KO animals had no expression. Blots were densitometrically normalized to β-actin and representative blots were cropped and presented

**Fig. 2:**
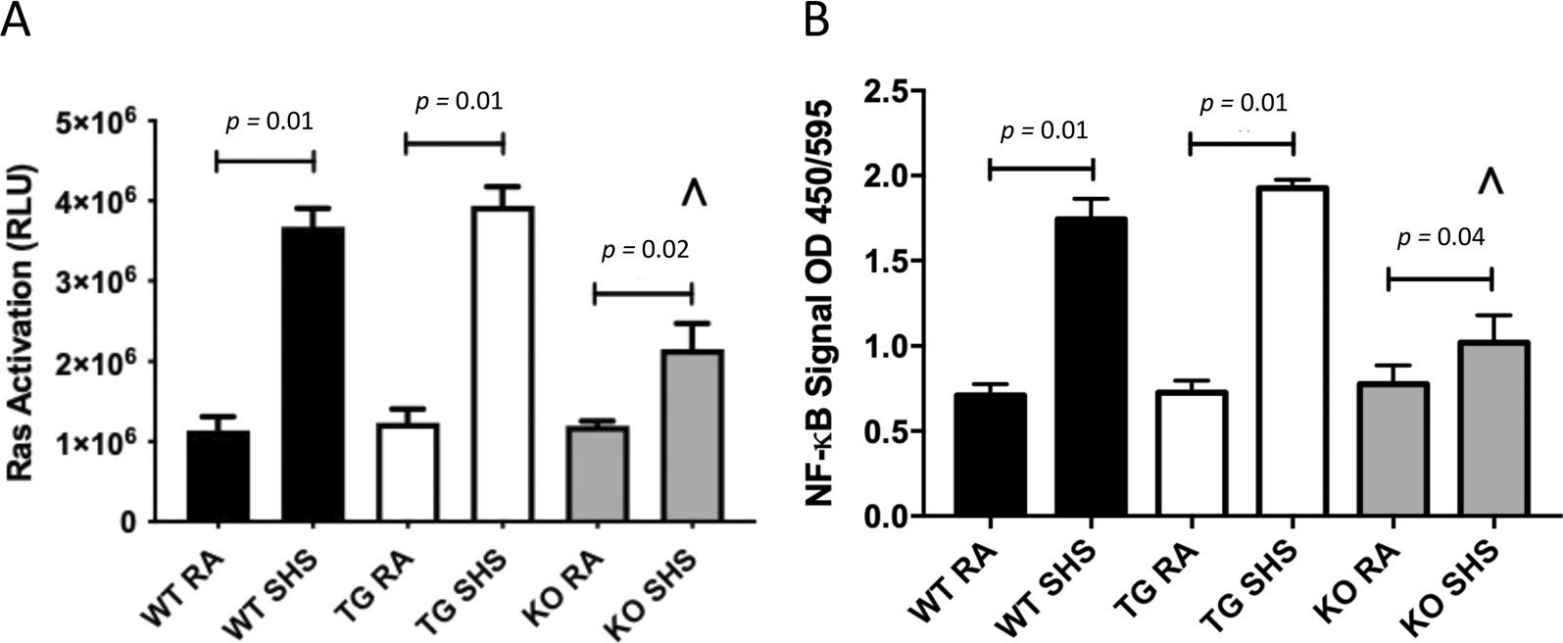
**A** Active Ras was significantly increased in WT (*p* = 0.01), RAGE TG (*p* = 0.01) and RAGE KO mice (*p* = 0.02) exposed to SHS compared to RA controls. Active Ras in SHS-exposed RAGE KO mice (^) was significantly less than SHS-exposed WT (*p* = 0.03) or RAGE TG (*p* = 0.02) mice. Data are representative of experiments (n = 6 mice per group). **B** Active NF-κB was significantly elevated in WT (*p* = 0.01), RAGE TG (*p* = 0.01), and RAGE KO (*p* = 0.04) mice exposed to SHS compared to RA counterparts. Active NF-κB in SHS-exposed RAGE KO mice (^) was significantly less than SHS-exposed WT (*p* = 0.03) or RAGE TG (*p* = 0.03) mice. Data are representative of experiments (n = 6 mice per group)

WT mice exposed to SHS experienced a significant increase in RAGE mRNA (Fig. 1A) and protein (Fig. 1B) compared to mice exposed to room air. RAGE mRNA and protein expression were also markedly increased in the lungs of RAGE TG mice following SHS exposure (Fig. 1A, B). As anticipated, RAGE expression was not detected in RAGE KO mice regardless of exposure (Fig. 1A, B). Quantification of RAGE signaling intermediates resulted in robust activation in all lines of mice following SHS exposure. The small GTPase Ras (a membrane-associated molecular mediator that perpetuates intracellular signaling [42]), was significantly increased in each of the three lines of mice (Fig. 2A). While Ras activation was also significantly increased by SHS in RAGE KO mice, the absence of RAGE resulted in significantly less active Ras following SHS exposure when compared to SHS-exposed WT or RAGE TG mice (Fig. 2A). We also observed significantly increased nuclear NF-κB activity in SHS-exposed WT, RAGE TG, and RAGE KO mice compared to room air (RA) controls (Fig. 2B). Notably, NF-κB activity in SHS-exposed RAGE KO mice was significantly decreased when compared to exposed WT or RAGE TG mice (Fig. 2B). Our data reveal RAGE induction by SHS and activation of known RAGE signaling intermediates including Ras and NF-κB by cells that coordinate cellular responses to exposure.

### Pulmonary inflammation and mechanics during SHS exposure

**Fig. 3:**
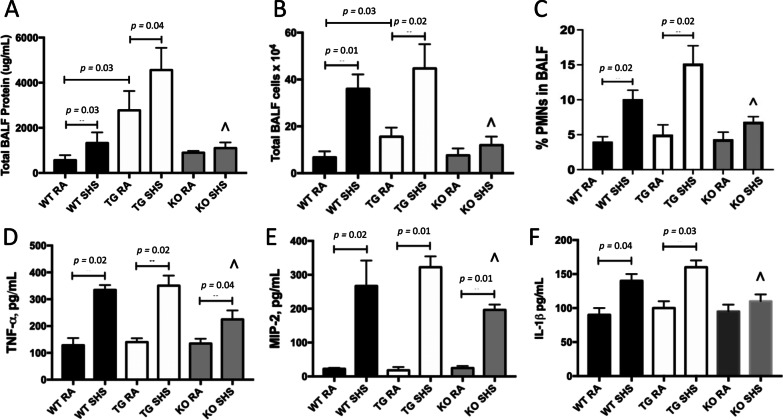
**A** Total protein in bronchoalveolar lavage fluid (BALF) was assayed using the BCA technique to demonstrate vascular permeability. In the absence of exposure, protein was significantly elevated in RAGE TG mice compared WT controls (*p* = 0.03). BALF protein was markedly elevated in WT (*p* = 0.03) and RAGE TG mice (*p* = 0.04) exposed to SHS compared to RA controls (n = 6 mice per group) and protein abundance was significantly decreased in SHS-exposed RAGE KO mice (^) compared to SHS-exposed WT (*p* = 0.05) or RAGE TG mice (*p* = 0.03). **B** Total BALF cells were significantly increased in unexposed RAGE TG mice compared WT mice (*p* = 0.03). Total BALF cellularity was also significantly increased in WT (*p* = 0.01) and RAGE TG mice (*p* = 0.03) exposed to SHS when compared to RA counterparts (n = 6 mice per group). Cellular abundance in BALF was significantly decreased in SHS-exposed RAGE KO mice (^) compared to SHS-exposed WT (*p* = 0.03) or RAGE TG mice (*p* = 0.03). C, PMNs were significantly increased in WT (*p* = 0.02) or RAGE TG mice (*p* = 0.02) exposed to SHS when compared to RA controls (n = 6 mice per group) and PMNs were decreased in SHS-exposed RAGE KO mice (^) compared to SHS-exposed WT (*p* = 0.04) or RAGE TG (*p* = 0.03) mice. D-F, Significantly more TNF-α (D), MIP-2 (E), and IL-1β (F) was secreted into BALF by mice exposed to SHS when compared to RA controls (n = 6 mice per group, *p* = 0.01–0.05 as indicated). The elaboration of TNF-α, MIP-2, and IL-1β were all significantly attenuated in RAGE KO mice (^) exposed to SHS compared to WT or RAGE TG mice exposed to SHS (n = 6 mice per group, *p* = 0.04–0.05)

Following exposure, we discovered evidence of pulmonary inflammation after assessing characteristics of bronchoalveolar lavage fluid (BALF). We measured total protein in BALF, an indirect assessment of vascular permeability, and observed elevated BALF protein in WT and RAGE TG mice following SHS exposure (Fig. 3A). However, there was no significant induction of BALF protein in SHS-exposed RAGE KO mice (Fig. 3A). Leukocyte cellularity was also elevated in BALF from WT and RAGE TG mice following exposure; however enhanced leukocyte extravasation, including PMNs, was not observed in RAGE KO mice following SHS exposure (Fig. 3B, C). Quantification of secreted TNF-α (Fig. 3D), MIP-2 (Fig. 3E) and IL-1β (Fig. 3F) resulted in significant increases in each cytokine’s abundance following SHS exposure compared to RA controls. While TNF-α and MIP-2 were each significantly elevated in RAGE KO mice following exposure (Fig. 3D, E), the expression of all three inflammatory mediators in RAGE KO was significantly attenuated compared to SHS-exposed WT or RAGE TG mice (Fig. 3D–F). Together, these BALF analyses demonstrate, at least in part, a RAGE-mediated response to SHS exposure.

**Fig. 4:**
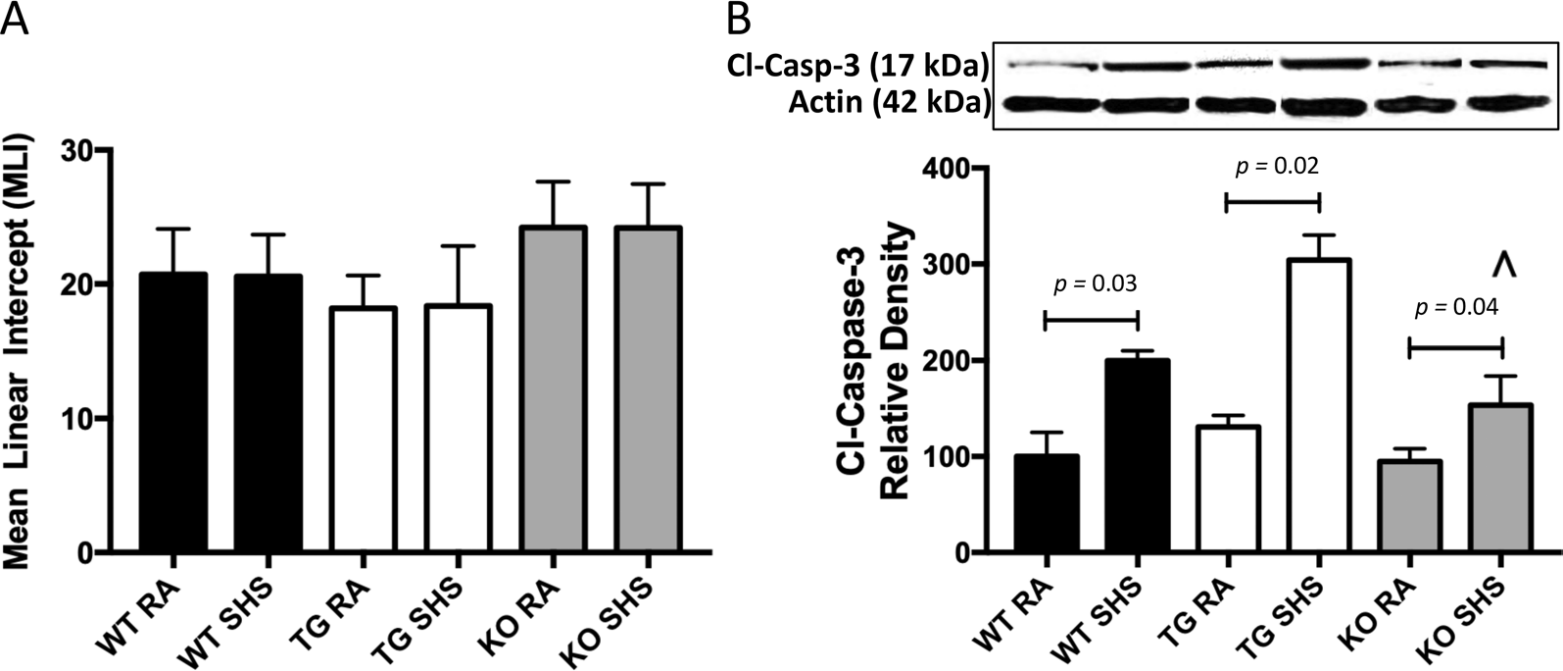
**A** There was no statistically significant alterations in the mean linear intercepts obtained from mice (n = 6 animals per group) regardless of exposure. **B** Increased activation of caspase-3 was detected in WT (*p* = 0.03), RAGE TG (*p* = 0.02), and RAGE KO (*p* = 0.04) mice exposed to SHS compared to RA controls. Cleaved caspase-3 was significantly decreased in SHS-exposed RAGE KO mice (^) compared to SHS-exposed WT (*p* = 0.04) or RAGE TG (*p* = 0.03) mice (^*p* ≤ 0.05). Blots were cropped and densitometrically normalized to β-actin and ratios of cleaved caspase-3/β-actin are presented

Representative lung histology revealed by H&E staining demonstrated no noticeable histopathology disturbances in RA-exposed WT, RAGE TG, or RAGE KO when compared to SHS-exposed counterparts (not shown). Such conclusions were supported by no significant differences in mean linear intercepts assessed in all three lines of mice exposed to RA or SHS (Fig. 4A). In accordance with our own data and numerous published reports, 4 weeks of SHS exposure is subthreshold in eliciting abnormal histology. However, our discovery that cleaved caspase-3, a mediator of parenchymal apoptosis, was increased in all three lines of mice exposed to SHS likely presages deleterious cellular loss should exposure continue (Fig. 4B). Tellingly, cleaved caspase-3 was significantly diminished in RAGE KO mice exposed to SHS when compared to SHS-exposed WT of RAGE TG mice (Fig. 4B).

**Fig. 5:**
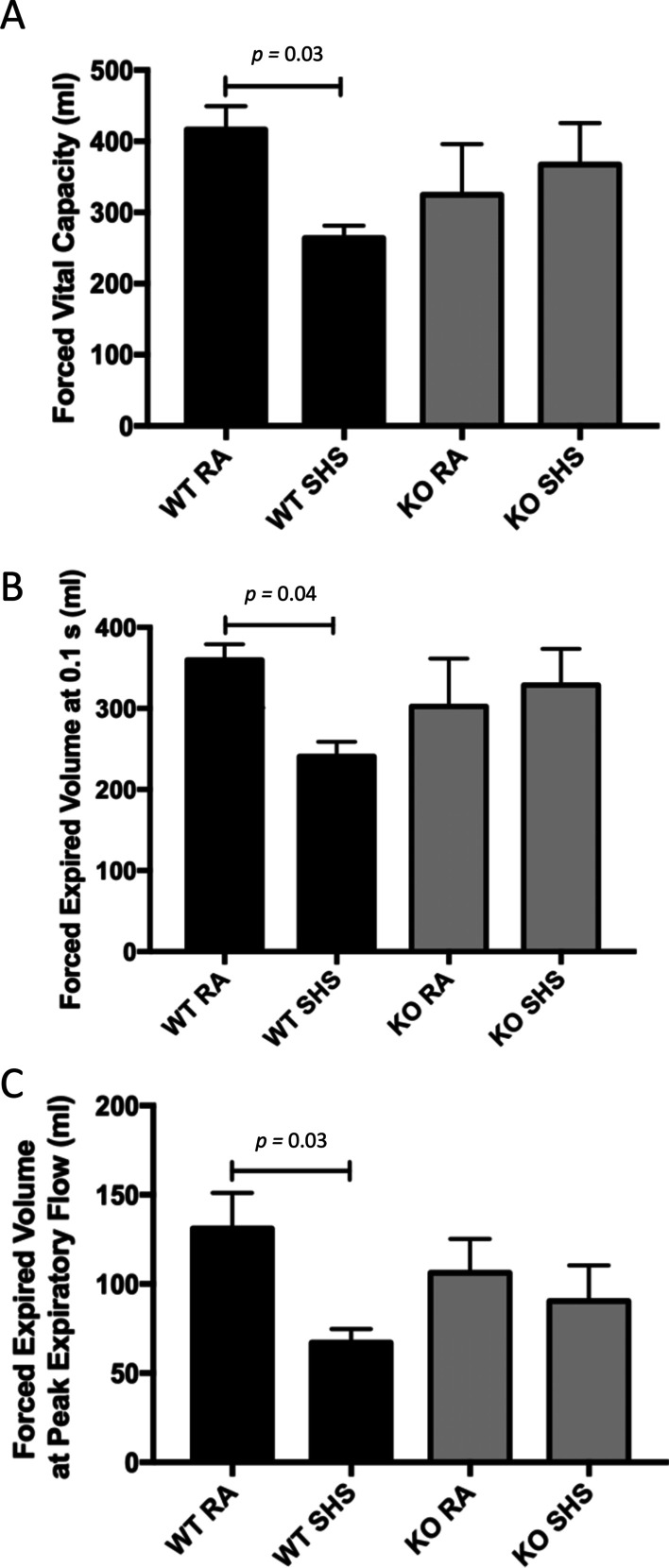
Lung physiology was evaluated in mice in order to determine alterations in mechanics. WT mice experienced significantly diminished FVC (Forced Vital Capacity, *p* = 0.03) (**A**) and FEV_0.1_ (Forced Expiratory Volume at 0.1 s, *p* = 0.04) (**B**) following SHS exposure. FEV at PEF (Peak Expiratory Flow) was also significantly diminished in WT mice exposed to SHS compared to RA controls (C, *p* = 0.03). RAGE KO animals were resistant to SHS-induced changes in FVC (**A**), FEV_0.1_ (**B**), and FEV at PEF (**C**). Evaluations were obtained in mice attached to the FlexiVent Instrument (Scireq) (n = 6 animals per group)

We evaluated lung physiology metrics in order to determine alterations in mechanics in the absence of tissue remodeling. WT mice experienced significantly diminished FVC (Forced Vital Capacity) (Fig. 5A) and FEV_0.1_ (Forced Expiratory Volume at 0.1 s) (Fig. 5B) following SHS exposure. FEV at PEF (Peak Expiratory Flow) was also significantly diminished in WT mice exposed to SHS compared to RA controls (Fig. 5C). RAGE KO animals were resistant to SHS-induced changes in FVC (Fig. 5A), FEV_0.1_ (Fig. 5B), and FEV at PEF (Fig. 5C) suggesting a physiologically protective role for RAGE during exposure to SHS. There were surprisingly no statistical differences between the groups when resistance, compliance or other gas volumes were screened after 30 days of exposure.

### Potential use of SAGEs to ameliorate the effects of RAGE during SHS exposure

**Fig. 6:**
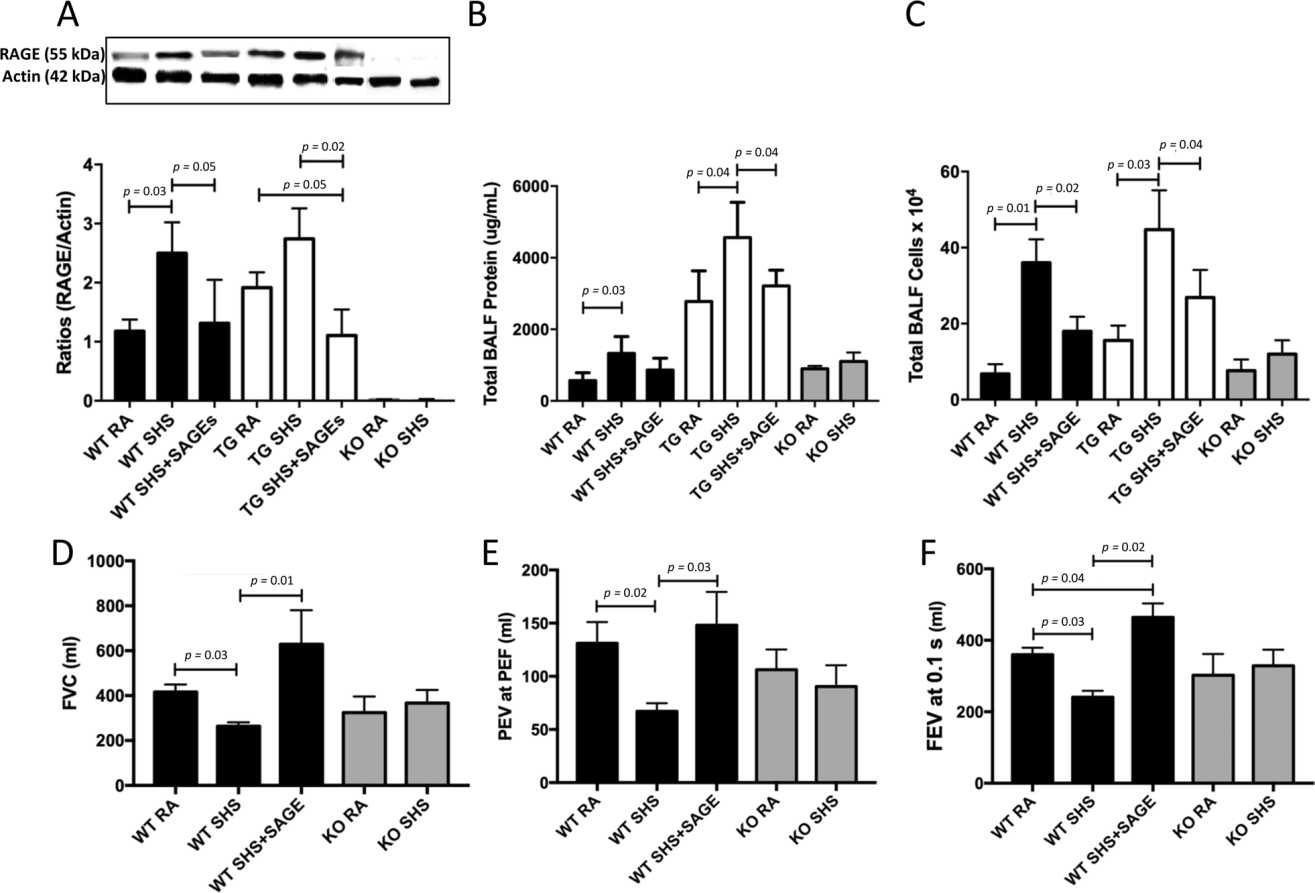
**A** SAGEs significantly reduced SHS-induced augmentation of RAGE protein in both WT (*p* = 0.03) and RAGE TG animals (*p* = 0.02) to near baseline and RAGE expression was not detected in RAGE KO mice. Blots were cropped and densitometrically normalized to β-actin and ratios of RAGE/β-actin are presented. **B** SAGEs decreased total BALF protein levels (B, *p* = 0.03–0.04 as indicated) and total BALF cellularity (C, *p* = 0.01–0.04 as indicated) in WT and RAGE TG exposed to SHS. Total BALF protein and leukocytes were unchanged in RAGE KO mice regardless of exposure (n = 6 mice per group). D-E, Exposure of WT mice to SAGEs prevented SHS-induced decreases in FVC (D, *p* = 0.01–0.03 as indicated) and PEV at PEF (E, *p* = 0.02–0.03 as indicated). FEV_0.1_ was significantly decreased following SHS exposure of WT mice and addition of SAGEs to exposed WT mice elicited an increase in FEV_0.1_ (F, *p* = 0.02–0.04 as indicated). Evaluations were obtained via the use of the FlexiVent Instrument (n = 6 animals per group)

Hyaluronic acid derivatives called semi-synthetic glycosaminoglycan ethers (SAGEs) inhibit RAGE ligand binding and have anti-inflammatory activity at low concentrations. We therefore sought to determine the extent SAGEs ameliorate SHS-induced anomalies in mice via the concomitant administration of SAGEs during exposure. We discovered that SAGEs inhibited SHS-induced augmentation of RAGE protein expression in both WT and RAGE TG animals (Fig. 6A). In fact, RAGE protein expression was returned to near baseline despite SHS exposure. As a reference, animals that lack RAGE expression (RAGE KO) did not express RAGE regardless of exposure (Fig. 6A). Availability of SAGEs also decreased total BALF protein levels (Fig. 6B) and total BALF cellularity (Fig. 6B) in WT and RAGE TG exposed to SHS. Total BALF protein and leukocytes in RAGE KO mice remained low and unchanged regardless of exposure (Fig. 6A, B).

We only evaluated WT (with and without SAGEs) and RAGE KO animals in order to confirm a role for RAGE in altering lung physiology. In the process, we discovered intriguing results in mice that did or did not express RAGE. Administration of WT mice with SAGEs prevented SHS-induced decreases in FVC (Fig. 6D) as well as PEV at PEF (Fig. 6E). FEV_0.1_ was significantly decreased following SHS exposure of WT mice (Figs. 5B and 6F). SHS + SAGEs in WT mice did not elicit a decrease in FEV_0.1_; however, FEV_0.1_ was significantly elevated (Fig. 6F).

## Discussion

With its unique apposition to the external environment, the respiratory system’s anatomy and physiology is responsible for coordinating immeasurable responses to inhaled materials. Anticipated responses to harmful entry of exogenous entities include the activation of cellular signaling axes that orchestrate immunomodulatory events required to maintain or repair affected tissues. In cases of pulmonary compromise stemming from tobacco smoke, persistent pulmonary inflammation results from activated immune cells, the secretion of inflammatory mediators, and tissue targeting pathways that hinder lung function. We discovered that RAGE was increased in WT mice and RAGE over-expressing transgenic mice following exposure (Fig. 1), an implication that this cell surface receptor likely functions in cellular responses to tobacco smoke [43]. RAGE was initially characterized as a pattern recognition receptor with ability to ligate AGEs and enhanced inflammatory end points following ligation [44]. AGEs are notably synthesized in inflammatory foci experiencing oxidative stress and research demonstrated that similar biochemical pathways mediated by Maillard reactions also occur in cases of tobacco smoke exposure and accumulate AGEs [18]. While the current research endeavor did not seek to identify specific RAGE ligands that induce signaling, AGEs and other ligands in tobacco smoke provide diverse ligand candidates that necessitate evaluation during enhanced RAGE pattern recognition. A recent publication by Sharma et al. reinforced the concept that inflammation coincides with RAGE ligands and that the AGE-RAGE signaling axis centrally impacts tobacco smoke-mediated lung disease [43].

Our observation that Ras and NF-κB were activated reveal that these molecules function during exposure and their activation is mediated, at least in part, by RAGE availability (Fig. 2). RAGE is further implicated as a key signaling modality because smoke-induced Ras and NF-κB activation was significantly attenuated in animals that lack RAGE expression. While both intermediates are functional in the progression of inflammatory lung diseases, there’s insufficient knowledge as to the association of downstream NF-κB genes and disease pathogenesis. Such gaps in knowledge may be more fully contemplated when considering the inflammatory cytokines we observed to be differentially regulated in the BALF from exposed mice. We found that TNF-α, MIP-2 (the mouse homologue of the human IL-8) and IL-1β were enhanced by SHS and significantly less expression was detected in SHS-exposed RAGE KO mice (Fig. 3). The elaboration of these specific cytokine were specifically supported by recent research conducted by Khan et al. that showed robust levels of these same mediators in waterpipe and cigarette smokers compared to non-smokers [45]. These molecules also coincided with elevated RAGE and En-RAGE detected in abundance in smoke-exposed groups [45]. TNF-α modulates the expression of diverse cytokines during inflammation, participates in adhesion molecule expression by endothelial cells during leukocyte diapedesis, and is highly expressed in the sputum of COPD patients [46]. MIP-2 (or IL-8) is highly expressed by patients with lung injury and via its role as a Th1 inflammatory cytokine, it functionally manages immune cell abundance [47]. IL-1β is also expressed in inflammatory conditions and it often partners with TNF-α in the coordination of cytokine elaboration and leukocyte chemotaxis [48]. We discerned leakiness of lung vasculature following exposure via enhanced BALF protein and cellularity. Zhou et al., demonstrated unique correlation between NF-κB family members and TNF-α, MIP-2, and IL-1β [49]. This research group suggested links between the activation of NF-κB genes and cytokines during lung inflammation. Our data showing amelioration of Ras/NF-κB signaling as well as BALF protein, leukocytes, and cytokines in exposed RAGE KO mice support these conclusions by implicating RAGE signaling in acute predisposing inflammation at the onset of lung disease (Figs. 2 and 3). Of course, additional chronic exposure studies should next be undertaken in order to clarify the impact of RAGE signaling on persistent lung pathobiology following smoke exposure.

Lung morphology assessed in each group were not distinguishable. These histological conclusions are likely a result of the timing of exposure, 4 weeks, and the accepted conclusion that lung remodeling in mouse models of pulmonary inflammation has not been detected following such an acute period. Quantification of linear intercepts in lung parenchyma from each group of mice confirmed no significant alteration (Fig. 4). Immunoblotting for cleaved caspase-3, a central cytosolic player in the mitochondrial intrinsic pathway of apoptosis, revealed elevated expression in each group of mice exposed to SHS (Fig. 4). This pro-apoptotic pathway has been identified as a key process of cell attrition during the pathogenesis of lung disease [50]. Research by Wu et al. revealed RAGE signaling as an axis the impacts apoptosis, invasion, and autophagy in the context of inflammation and cancer [51]; revealing the pathway as a contributor to deleterious cell turnover. Despite no observable parenchymal alterations after 4 weeks of exposure, we demonstrate that caspase-3 mediated apoptosis is underway and remodeling may only be observable after prolonged periods of SHS exposure. Consistent with typical patterns of spirometry assessed in human patients, lung physiology metrics in exposed mice demonstrated consistent deficits of lung function (Fig. 5). Diminished FVC, FEV_0.1_ and FEV at PEF are each associated with inflammatory lung injury and the degree of reversibility in these metrics is often relied upon when characterizing potential therapies [52]. We discovered no SHS-induced depressions in FVC, FEV_0.1_ and FEV at PEF obtained from RAGE KO mice.

While not all metrics were able to be screened, our data demonstrate that SAGEs markedly reduce RAGE abundance, pulmonary inflammation and physiological mechanics in the lungs of mice exposed to SHS (Fig. 6). We specifically observed that prophylactic administration of SAGEs reduced RAGE expression in WT and RAGE TG animals to baseline despite genetic up-regulation programs of SHS exposure. Evaluations of BALF protein and cellularity revealed that WT animals exposed to SHS and SAGEs were not different than RAGE KO BALF regardless of SHS. These observations firmly show that RAGE targeting by SAGEs, at least in these end-points, is not different than RAGE KO animals exposed to SHS. Further, while FVC, FEV_0.1_ and FEV at PEF volumes were not different in RAGE KO mice exposed to either RA or SHS, each volume assessed in mice with SHS and SAGEs returned to or exceeded the levels observed in WT mice exposed to RA. Additional parameters of lung function including resistance and compliance were not different among the groups, potentially due to the acute nature of the exposure period. These compelling observations add to the narrative that existing and emerging therapies for lung inflammation may be aided by solutions involving inflammation-modulating glycosaminoglycans (GAGs).

This current research does not attempt to model COPD pathogenesis, rather model RAGE effects during inflammation caused by acute exposure. In particular, this research provides a significant step forward in the concept that RAGE availability modulates SHS-induced inflammation. While use of RAGE KO animals initially demonstrated a means of diminishing inflammation [25], this project expanded RAGE expression dynamics via conditional transgenic mouse models and considered a small molecular inhibitor of RAGE signaling. It therefore adds to the narrative that targeting RAGE ameliorates smoke-induced airway inflammation and downregulates immune-inflammatory signaling networks [53]. This proof-of-concept approach shows that RAGE abrogation and the utilization of RAGE-modulating SAGEs may feasibly be effective in lessening symptoms associated with smoke exposure. However, limitations to these in vivo studies remain in terms of animal models and timing. Inflammatory lung diseases generally are heterogeneous afflictions and little consensus exists as to which mouse models are most effective in mimicking their myriad symptoms. A more complete experimental approach is required so that additional inflammatory readouts, complete with tissue remodeling progression, can be pursued over a chronic time course. Such an undertaking could also further delineate prophylactic and/or therapeutic utility.

## Conclusion

**Fig. 7:**
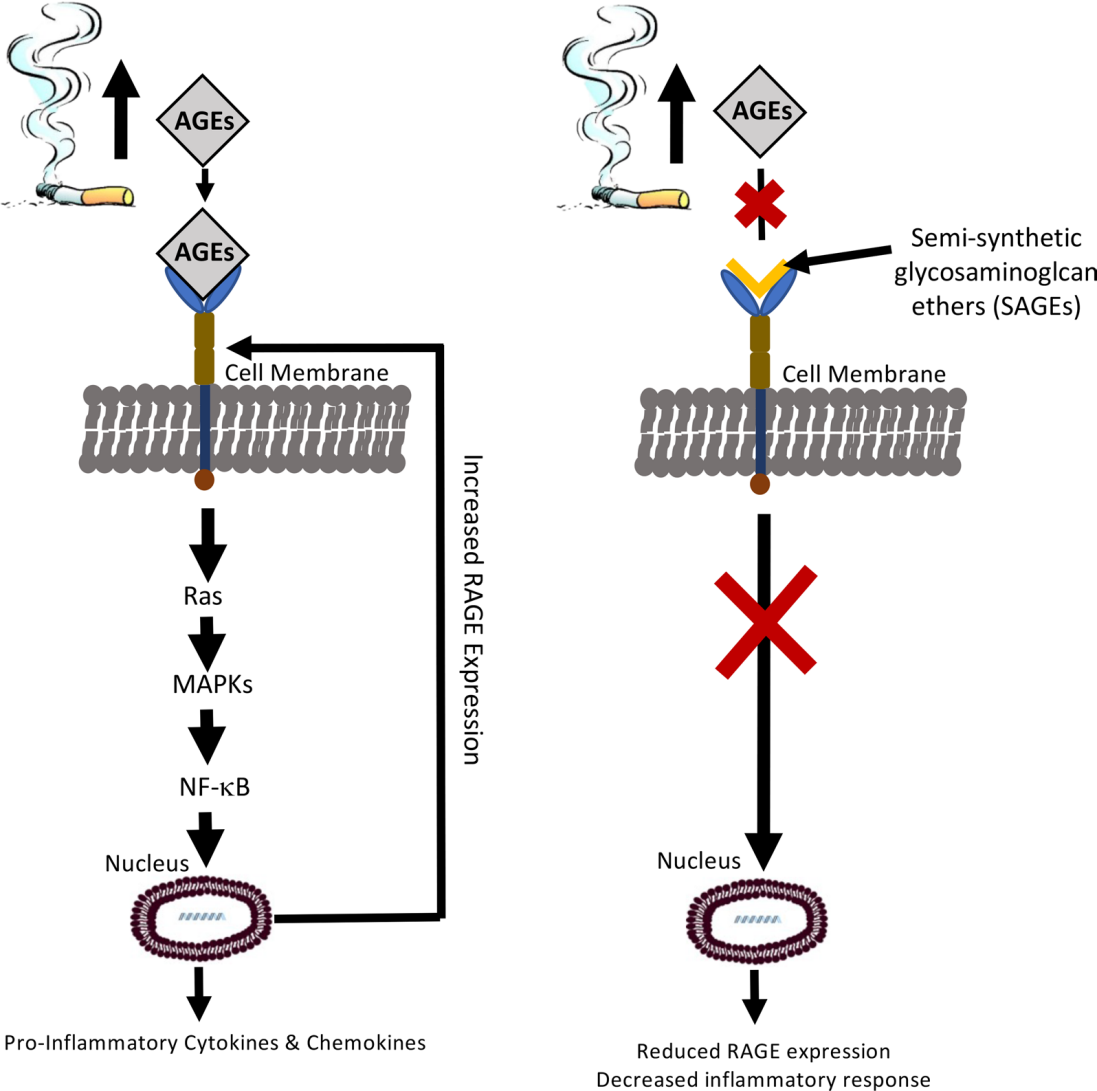
An initial working model whereby RAGE augmentation during SHS exposure coincides with pro-inflammatory signaling and RAGE targeting ameliorates inflammatory responses

Tobacco smoke exposure up-regulates RAGE expression and elicits inflammatory responses. The data presented here demonstrate that RAGE augmentation during exposure coincides with inflammatory signaling and that RAGE targeting ameliorates characteristics of lung inflammation (Fig. 7). Furthermore, SAGEs, novel small molecules that prevents RAGE-ligand binding, may be effective in lessening tobacco smoke-induced pulmonary inflammation.

## Data Availability

All data are presented within the article. Data and other materials are available from the corresponding author on reasonable request.

## Abbreviations

AGEs: Advanced glycation end-products
BALF: Bronchoalveolar lavage fluid
COPD: Chronic obstructive pulmonary disease
HA: Hyaluronic acid
H&E: Hematoxylin and eosin
MAPK: MAP Kinases
PN: Post-natal day
RAGE: Receptors for advanced glycation end-products
SAGEs: Semi-synthetic glycosaminoglycan ethers
SHS: Secondhand smoke
TG: Transgenic
